# PARTNERING TO IMPROVE CARE: STRENGTHENING THE FAMILY CAREGIVER AND HOME CARE WORKER RELATIONSHIP

**DOI:** 10.1093/geroni/igad104.1522

**Published:** 2023-12-21

**Authors:** Kezia Scales, Jessica King, Emily Dieppa

**Affiliations:** PHI, New York City, New York, United States; PHI, New York City, New York, United States; PHI, New York City, New York, United States

## Abstract

Family caregivers and home care workers provide essential daily care for millions of older adults and individuals with disabilities across the United States—and together they constitute the backbone of home and community-based services (HCBS). In many cases, these unpaid and paid caregivers work closely together and share common knowledge, skills, experiences, and challenges. Yet the overlap and relationship between them remains relatively understudied. This session will describe the importance of strengthening the family caregiver/home care worker dyad and discuss promising developments in research, policy, and practice. We will then highlight a pilot training intervention designed to improve the shared knowledge and interpersonal competencies of family caregivers and home care workers. The intervention— Partnering to Improve Care: Direct Care Workers and Families—was developed by PHI (a national nonprofit research, public education, and consulting organization) in partnership with the Florida Pioneer Network, Home Instead, and Florida State University through the North and Central Florida Geriatrics Workforce Enhancement Partnership (GWEP). Informed by the PHI Coaching Approach®, the intervention consists of five parallel online training modules for family caregivers and for home care workers that utilize adult learner-centered teaching methods and reflect input from home care stakeholders. The modules portray common scenarios that family caregivers and home care workers face and aim to build communication and relationship skills that facilitate collaborative problem-solving and the provision of person-centered care. We will present key findings on the feasibility and acceptability of this intervention, discuss next steps in this research, and highlight broader policy and practice recommendations.

